# Brain tumour segmentation based on an improved U-Net

**DOI:** 10.1186/s12880-022-00931-1

**Published:** 2022-11-18

**Authors:** Ping Zheng, Xunfei Zhu, Wenbo Guo

**Affiliations:** 1 Anhui University of Science and Technology, Anhui, 232001 China; 2 Yunnan University, Yunnan, 650500 China

**Keywords:** Brain tumour, Dice loss, Encoding–decoding, HDC, Segmentation, U-Net

## Abstract

**Background:**

Automatic segmentation of brain tumours using deep learning algorithms is currently one of the research hotspots in the medical image segmentation field. An improved U-Net network is proposed to segment brain tumours to improve the segmentation effect of brain tumours.

**Methods:**

To solve the problems of other brain tumour segmentation models such as U-Net, including insufficient ability to segment edge details and reuse feature information, poor extraction of location information and the commonly used binary cross-entropy and Dice loss are often ineffective when used as loss functions for brain tumour segmentation models, we propose a serial encoding–decoding structure, which achieves improved segmentation performance by adding hybrid dilated convolution (HDC) modules and concatenation between each module of two serial networks. In addition, we propose a new loss function to focus the model more on samples that are difficult to segment and classify. We compared the results of our proposed model and the commonly used segmentation models under the IOU, PA, Dice, precision, Hausdorf95, and ASD metrics.

**Results:**

The performance of the proposed method outperforms other segmentation models in each metric. In addition, the schematic diagram of the segmentation results shows that the segmentation results of our algorithm are closer to the ground truth, showing more brain tumour details, while the segmentation results of other algorithms are smoother.

**Conclusions:**

Our algorithm has better semantic segmentation performance than other commonly used segmentation algorithms. The technology we propose can be used in the brain tumour diagnosis to provide better protection for patients' later treatments.

## Background

Tumours have always been a feared disease; brain tumours have an incidence rate of 1.5% and an alarming 3% mortality rate in the population and are feared because of their extremely high incidence and mortality rate [1]. A brain tumour is a cancer of the brain tissue that is formed when the brain tissues become cancerous or metastasize to other tissues in the skull. With medical imaging technology development, imaging technology has gradually been applied to tumour detection. Initially, computed tomography (CT) technology was used for detection, but with the development of magnetic resonance physics and then the combination with the theory and technology of digital image reconstruction, magnetic resonance imaging (MRI) is slowly taking shape because it does not cause ionizing radiation damage to the body, and many imaging parameters have gradually become the mainstream of medical brain tumour detection [2]. However, most of the current clinical brain tumour diagnoses are based on the clinician's experience. The method of manually segmenting, diagnosing and annotating tumour images is inefficient and demanding for image analysts, and it is easy to miss the best treatment window for patients [3]. Therefore, how to efficiently diagnose brain tumour images and reduce image diagnostic error has become a research direction for many researchers. Currently, deep learning-based intelligent algorithms are widely used in brain tumour analysis tasks, and CNNs are adopted by researchers for their good segmentation performance and the convenience of feature extraction [4]. However, CNNs are prone to computational redundancy when processing a large number of dense images [5]. Therefore, FCN [6], U-Net [7] and other derived algorithms based on CNNs have been proposed. However, many brain tumour segmentation algorithms still have many problems, such as the segmentation accuracy and recognition accuracy of the algorithms are not high enough, and the attention to detail is not sufficient. In this paper, we propose an improved segmentation network based on U-Net to solve these problems using a tandem encoding–decoding model and proposing a new loss function to increase the weight of samples that are difficult to classify. The experimental results show that our proposed method outperforms several other commonly used derived models based on CNNs in terms of segmentation performance and tumour recognition accuracy.

Performing image segmentation is a key problem in the computer vision (CV) field, and image segmentation generally includes semantic segmentation and instance segmentation [8]. Brain tumour segmentation in this paper uses semantic segmentation. Evaluating the segmentation ability of the semantic segmentation model needs to focus not only on the overall image segmentation but also on edge segmentation. Therefore, how to design the segmentation algorithm becomes important, and different researchers have proposed different methods for segmentation algorithm research. With the rise of neural network models and the development of deep learning, segmentation networks based on deep learning have been rapidly developed and applied. Starting from the concept of neural networks proposed by Le Cun, neural networks have been developed rapidly, and various neural network structures have begun to emerge slowly, such as AlexNet [9], VGG [10], and ResNet [11]. Although these networks have advantages in the image recognition and prediction field, the advantages in accurate semantic image segmentation are not as obvious. To change this situation, Shelhamer et al. proposed FCN, applying FCN to semantic image segmentation [6]. They achieved segmentation by replacing the fully connected layers of the network with convolutional layers, and the results showed that the semantic image segmentation outperformed the other convolutional neural networks (CNNs). The reason is that full convolutional networks (FCNs) require a high data volume, and such brain tumour images are relatively few and precious in medicine. To solve this problem, Ronnerberger et al. modified the fully convolutional network by adopting transposed convolution, upsampling, and fusing context features and detail features to form U-Net, which can obtain enough data features with few brain images, and the segmentation effect is significantly better than that of a fully convolutional network (FCN). However, there are still problems of incomplete information and low segmentation accuracy when performing brain tumour segmentation. To solve the remaining problems of the U-Net network, Alom proposed a recursive neural network based on U-Net and a recursive residual convolution neural network based on the U-Net model [12]. Zhang et al. used the U-Net extended path to design residual connections and proposed a depth residual U-Net for image segmentation [13]. Milletari proposed a 3D U-Net model, which uses a 3D convolution kernel to expand the original U-Net structure and then adds residual units to further modify the original U-Net structure [14]. Salehi used an auto context algorithm to enhance U-Net to improve the segmentation effect [15]. Zhou et al. used the nesting method to replace the original connection method [16]. Wanli Chen, Yue Zhang et al. proposed a stacked U-Net with a bridging structure to address the problem of increasing training difficulty as the number of layers of the network increases [17]. The above segmentation model can only segment images but cannot grade segmented tumours. To achieve this clinical need, Mohamed A. Naser and M. Jamal Deen first used the trained segmentation model and MRI images for mask generation and then used a densely connected neural network classifier to classify the tumour [18].

## Materials and methods

### Brain tumour MRI images

The dataset was obtained from the Kaggle open source database "Brain Tumour MRI Image Classification", which contains three main types of brain tumours: glioma tumours, meningioma tumours, and pituitary tumours. The sample size of the training set containing brain tumours was 2,475, and the sample size of the test set was 289. First, we screened the dataset and selected sections with brain tumours in the sample as our experimental dataset. Then, image enhancement was carried out, and the sample size of the enhanced dataset was 2,624. Finally, manual labelling of the enhanced sections was completed with the help of graduate students from the medical college. The labelled images were reassigned according to the 10:1 ratio of the training set and test set.

### Encoding module and decoding module

The SCU-Net proposed in this paper consists of two encoding modules and two decoding modules. The VGG16 net and HDC model chosen in this paper are used as the basic framework of the encoding module. Most neural network models use maximum pooling to reduce the network volume and highlight the main features when performing feature extraction. This method may ignore the segmentation details and lose the spatial location information of the main features. However, brain tumour cutting requires accuracy to the millimetre or micron level, which requires us to obtain more detailed features and minimize feature loss in training. We know that larger convolutional kernels may capture more positional information than smaller convolutional kernels because a larger receptive field can better resolve positional information [19]. Therefore, we chose hybrid dilated convolution (HDC) [20]. The HDC module can increase the receptive field, improve the ability to obtain global information, and alleviate the grid problem of dilated convolution. The hybrid dilated convolution operator is as follows:

1 $$\begin{gathered} \left( {F*_{{l_{1} }} k} \right)({\mathbf{p}}) = \sum\limits_{{{\mathbf{s}} + l_{1} {\mathbf{t}} = {\mathbf{p}}}} F ({\mathbf{s}})k({\mathbf{t}}) \hfill \\ \left( {F*_{{l_{2} }} k} \right)({\mathbf{p}}) = \sum\limits_{{{\mathbf{s}} + l_{2} {\mathbf{t}} = {\mathbf{p}}}} F ({\mathbf{s}})k({\mathbf{t}}) \hfill \\ \vdots \hfill \\ \left( {F*_{{l_{n} }} k} \right)({\mathbf{p}}) = \sum\limits_{{{\mathbf{s}} + l_{n} {\mathbf{t}} = {\mathbf{p}}}} F ({\mathbf{s}})k({\mathbf{t}}) \hfill \\ \end{gathered}$$

In the formula, $l_{1}$ to $l_{n}$ are hybrid dilated convolution operators with different dilation factors, and the maximum common divisor of L1 to Ln is not greater than 1. $F:{\mathbb{Z}}^{2} \to {\mathbb{R}}$ is a discrete function. $\Omega_{r} = [ - r,r]^{2} \cap {\mathbb{Z}}^{2}$ and $k:\Omega_{r} \to {\mathbb{R}}$ are discrete filters.

**Fig. 1:**
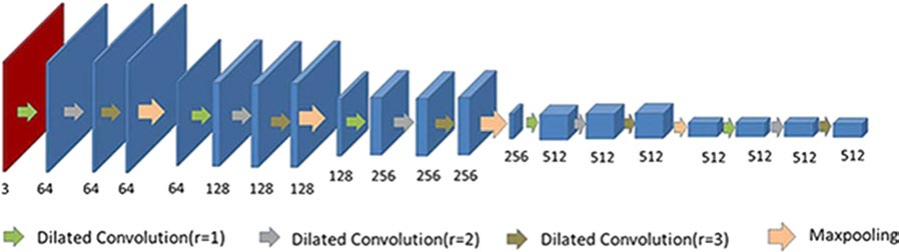
SCU-Net backbone network

We use multiple convolution blocks with different expansion rates and connect them in the same way. Each hybrid dilated convolution and a ReLU function form an HDC module, and three HDC modules with different expansion rates form an HDC module group. We replace the ordinary convolution of each layer in the two VGG encoding modules with an HDC module group. The dilation rates are selected as 1, 2 and 3 to satisfy the formula, $M_{i} = \max \left[ {M_{i + 1} - 2r_{i} ,M_{i + 1} - 2\left( {M_{i + 1} - r_{i} } \right),r_{i} } \right]$, where $M$ represents the maximum distance between two nonzero values. The distribution of the dilation rate into the sawtooth wave pattern helps the top layer obtain more information while keeping the receiving field unchanged compared with the original configuration in which the dilation rate is the same [20]. The encoding network structure is shown in Fig. 1, which is divided into 5 layers. Each layer is composed of a group of dilated convolution blocks and maximum pooling. Each group of dilated convolution blocks is composed of three 3 × 3 dilated convolutions (the dilation rate of dilated convolution is 1, 2, and 3), three BN layers, and three ReLU functions. The input image is 512 × 512 × 1, and the image under the RGB channel is obtained through conversion. Then, a 64-channel 512 × 512 feature map is obtained through a dilated convolution block with a convolution kernel size of 3 × 3. After the first layer HDC module group and downsampling, a feature map with 128 channels of 256 × 256 size is obtained. Through the second layer HDC module group and downsampling, a 128 × 128 feature graph of 256 channels is obtained. Through the third layer HDC module group and downsampling, the 512 channel number and 64 × 64 size feature graph are obtained. Through the HDC module group and downsampling in the fourth layer, the 512 channel number and 32 × 32 size feature map are obtained. Then, these feature graphs are introduced into the decoding network and the second encoding network. SCU-Net using the HDC module can better solve the chessboard effect of dilated convolution, increase the receptive field of the coding network, and improve the capture of the details of the input image, the edge information, and the relative position information of the tumour in the input image.

The decoding module of SCU-NET is composed of multiple convolutions and transposed convolutions. The information of the next layer is first expanded by a 3 × 3 transposed convolution of pixels and then concatenated and convolved with the feature map of the same layer. If it is in the second decoding net, it also needs to be spliced and convolved with the image of the same layer after the first decoding, then upsampled and concatenated with the upper layer, and repeatedly upsampled, concatenated and convolved, thus continuously recovering pixels until the pixels are the same as the original image, and finally convolved again, so that the number of channels is the number of the desired classification. The specific concatenation method and other details are introduced in Series Section and Concatenation.

Such a decoding method combining all the information from the previous layers and the serial network can obtain more contextual semantic information and effectively extract the feature information of the same layers many times and then obtain more accurate prediction results and segmentation capability.

### Series section and concatenation

The complete architecture of SCU-Net connects two encoding–decoding modules in series and then bridges each layer for feature sharing purposes. Several existing works show that the interactions between global features or contexts help to perform semantic segmentation, and we experimented with two bridging approaches, a pixel summing approach and a channel concatenation approach. Finally, we used the concatenation approach to connect the different modules.

**Fig. 2:**
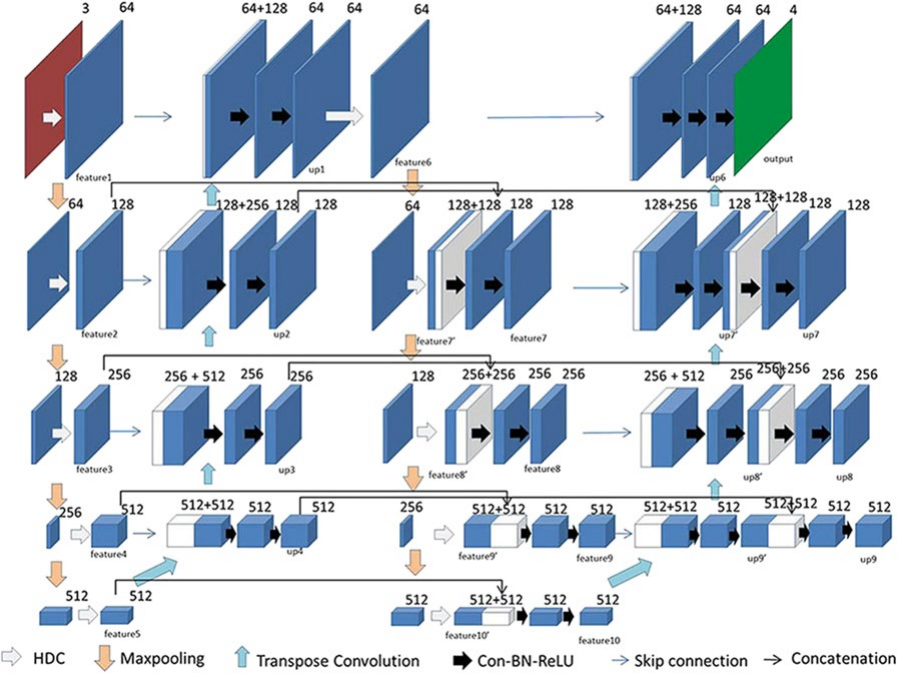
Overall architecture of SCU-Net

Figure 2 shows the overall architecture of SCU-Net, which is composed of two encoding–decoding structures in series and has many concatenation operations between two series to add the interactions between two encoding–decoding nets. As shown in Fig. 2, we defined the feature obtained from the input image through the HDC module group with a convolution kernel size of 3 × 3 in the first layer of the first encoding structure as feature 1 and the feature obtained from the image downsampling in the previous layer through the HDC module group with a convolution kernel size of 3 × 3 in the second layer as feature 2, and the other feature maps are defined in the same order. We defined the matrix obtained by transposed convolution with a convolution kernel size of 4 × 4 and convolution operation with a convolution kernel size of 3 × 3 in the first layer of the first decoding structure as up1, and the matrix named up2 is finally obtained by concatenating with the image upsampled in the next layer and two convolutions in the second layer. The other matrices after upsampling are defined in the same order. In the first layer of the second encoding structure, up1 passes through the HDC module group with a kernel size of 3 × 3 to obtain feature6. In the second layer, feature7' is obtained through max-pooling with a kernel size of 2 × 2 and the HDC module group with a kernel size of 3 × 3. In the third layer, feature8' is obtained through downsampling of the upper layer feature and passing through the HDC module group with a kernel size of 3 × 3. The definitions of other layers are similar to those above. In the first layer of the second decoding structure, the upsampling results of the next layer are skip-connected to feature 6 and then convolved twice with a kernel size of 3 × 3 to obtain up6. In the second layer, the image obtained by upsampling the next layer and two convolutions with a kernel size of 3 × 3 is called up7'. In the third layer, the image obtained by upsampling the next layer and two convolutions with a kernel size of 3 × 3 is called up8’. The definitions of other layers are similar to those above.

The two serial encoding–decoding structures are connected using many concatenations. The specific connection mode is as follows: feature 5 and feature10' of layer 5 perform a concatenation operation, and then the number of channels of the image is recovered by two 3 × 3 convolutions to obtain feature10 with 512 channels. Feature 4 and feature 9' of the fourth layer perform concatenation and then recover the number of channels by two 3 × 3 convolutions to obtain feature 9 with 512 channels. Up4 and up9' perform concatenation and then recover the number of channels by two 3 × 3 convolutions to obtain up9 with the channel of 512. Feature3 of the third layer and feature8' perform concatenation and then recover the number of channels by two 3 × 3 convolutions to obtain feature8 with the channel of 256. Up3 and up8' perform concatenation and then recover the number of channels by two 3 × 3 convolutions to obtain up8 with the channel of 256. Feature 2 of the fourth layer and feature 7' perform concatenation and then recover the number of channels by two 3 × 3 convolutions to obtain feature 7 with 128 channels. Up2 and up7' perform concatenation and then recover the number of channels by two 3 × 3 convolutions to obtain up7 with 128 channels.

Unlike stacked U-Net, SCU-Net directly uses the output of the previous encoding–decoding structure as the input of the next encoding–decoding structure, maximizing the use of the image information after the previous decoding, avoiding structural redundancy, and being more conducive to the extraction of key information and mining. Linking the same layers of two encoding–decoding structures through concatenation can deepen the interconnection between the two encoding–decoding structures, strengthen semantic information sharing between two encoding–decoding networks, reduce feature redundancy, and accelerate learning speed. In addition, performing secondary feature extraction of the previous information can obtain richer semantic information and thus can obtain a better segmentation effect.

### Focal dice loss function

This paper proposes a new loss function called focal Dice loss. It combines the advantages of focal loss and log-cosh Dice loss and improves the disadvantage that log-cosh Dice loss can only assign the same weight to all samples so that the loss function can assign more weight to the samples that are difficult to segment to enhance the model's learning ability for the sample.

The Dice coefficient is a commonly used performance metric for segmentation tasks and thus has also been modified as a loss function to obtain higher segmentation performance, but since the Dice coefficient loss is a nonconvex function, the training process may not achieve the desired results; therefore, Shruti Jadon et al. proposed the log-cosh Dice loss to solve the problem of the nonconvex loss function by adding smoothing through Lovsz expansion [21]. The formula is as follows:

2 $$\cosh x = \frac{{e^{x} + e^{ - x} }}{2}$$

3 $$L_{lc - dce} = log(cosh( \, DiceLoss \, ))$$

Although log-cosh Dice loss accounts for the nonconvexity problem, the same loss coefficient is used for different segmentation samples. It is not conducive to the network's learning of segmentation samples with different training difficulties. We improve the log-cosh Dice loss so that it can achieve adaptive changes for different samples by reducing the weights of easily segmented samples and adding the weights of difficult segmented samples, making the model focus more on the hard-to-segment samples during training. In addition, the improved log-cosh Dice loss and focal loss are combined so that the loss function can focus on both model classification ability and model segmentation ability. The loss function we propose is called focal Dice loss, as shown in formula (4). $\omega_{1}$ and $\omega_{2}$ are used to adjust the weights between the focal loss and the improved log-cosh Dice loss.$\left( {DiceLoss} \right)^{\gamma }$ adaptively adjusts the weight of log-cosh Dice loss in the focal Dice loss. If the log-cosh Dice loss of the current sample is large, which means the segmentation effect is poor, then $\left( {DiceLoss} \right)^{\gamma }$ increases. If the log-cosh Dice loss of the current sample is small, which means that the segmentation effect is good, excessive weight is not needed, and the formula automatically decreases $\left( {DiceLoss} \right)^{\gamma }$ to weaken the dependence on the log-cosh Dice loss.

4 $$\begin{aligned} & Focal - Dice Loss = \omega_{1} (FocalLoss) \\ & \quad + \omega_{2} \left( {DiceLoss} \right)^{\gamma } log(cosh( \, DiceLoss \, )) \\ \end{aligned}$$

### Evaluation metrics

TO verify the scientific nature of the research in this paper, the evaluation index of image semantic segmentation is selected for evaluation. It includes MIoU, MPA, precision, Dice, accuracy, Hausdorff, and ASD.

Mean-intersection-over-union (MIoU), which is the average of the ratio of intersection and merge of all categories calculated and is a common standard metric function for semantic segmentation, is as follows:

5 $$MIoU = \frac{1}{k + 1}\sum\limits_{i = 0}^{k} {\frac{{p_{ii} }}{{\sum\limits_{j = 0}^{k} {p_{ij} } + \sum\limits_{j = 0}^{k} {p_{ji} } - p_{ii} }}}$$

where $k$ denotes the category, $i$ denotes the true value, $j$ denotes the predicted value, $p_{ij}$ denotes the number of pixels that predict $i$ to $j$, $p_{ji}$ denotes the number of pixels that predict $j$ to $i$, and $p_{ii}$ denotes the number of correctly predicted pixels.

MPA is the average of PA. PA denotes the ratio of the number of pixels correctly predicted to the total number of pixels. MPA denotes the cumulative averaging of each category, as follows:

6 $$MPA = \frac{1}{k + 1}\sum\limits_{i = 0}^{k} {\frac{{p_{ii} }}{{\sum\limits_{j = 0}^{k} {p_{ij} } }}}$$

In the formula, TP indicates that both the predicted and real values of the sample are positive, FP indicates that the predicted value of the sample is positive and the real value of the sample is negative.

The Dice coefficient is a similarity measurement function that judges the similarity of two samples. The greater the similarity between the two input sets, the greater the Dice coefficient, and the more accurate the segmentation model. It is one of the important indicators for image segmentation, and the specific formula is as follows:

7 $$\, Dice \, = \frac{2TP}{{2TP + FP + FN}} = \frac{2|X \cap Y|}{{|X| + |Y|}}$$

Y represents the predicted target, and X represents the ground truth.

Accuracy is our most common evaluation metric, which refers to the ratio of the number of samples that are scored correctly to the number of all samples, and the higher the accuracy is, the better the model effect.

8 $${\text{Accuracy }} = \frac{TP + TN}{{TP + TN + FP + FN}}$$

TN indicates that both the predicted and real values of the sample are false. FN denotes that the predicted value of the sample is false and the real value of the sample is true.

The Hausdorff distance is used to calculate the distance between the real value boundary and the predicted value boundary, and a smaller distance between the two indicates a higher segmentation accuracy. The specific formula is as follows:

9 $$HD = \max \left\{ {\vec{d}_{H} (A,B),\vec{d}_{H} (B,A)} \right\}$$

The average surface distance (ASD) is the average of all distances from a point on the object boundary to the GT boundary, and the specific formula is as follows:

10 $$ASD = \frac{1}{{\left| {B_{AS} } \right|}}\sum\limits_{{x \in B_{AS} }} d \left( {x,B_{GT} } \right)$$

## Result

The experiment is divided into three parts. First, we modify the convolution part of the UNetVGG16 backbone network by replacing the original convolution block with an HDC block and compare it with the original UNetVGG16 model. Then, we modify the loss function of UNetVGG16, replace the original cross-entropy loss function with our proposed focal Dice loss, and compare it with the original UNetVGG16 model. Finally, we compare the performance capability of our proposed SCU-Net with several commonly used semantic segmentation models on our brain tumour dataset.

### Experimental environment

The framework used in the experiments is PyTorch, and the specifications of the machine are as follows: graphics card: Tesla P40; video memory: 22 G; CPU: Intel(R) Xeon(R) CPU E5-2680 v4; memory: 440 G, cores: 56. We optimize the model using the Adam optimizer and adjust the learning rate using the cosine annealing function by setting the cosine annealing function.

### UNetVGG16 + HDC

To verify the effectiveness of the UNetVGG16 model using the HDC module, we compare UNetVGG16 with the HDC added with UNetVGG16. We replace the convolution in the original UNetVGG16 with the HDC module, train 25 epochs, and set the dilation rates of the three dilated convolutions of the HDC module to *r1* = 2, *r2* = 3, *r3* = 4; the *padding* to *padding1* = 2, *padding2* = 3, *padding3* = 4. The experimental results are shown in Table 1. As shown in Table 1, the results of HDC under each index are better than those of UNetVGG16. The index with the most improvement is Hausdorf95 with an increase of 27.08 percentage points, followed by MIoU with an increase of 9.51 percentage points.

**Table 1:** Performance of UNetVGG16 after adding different modules

Networks \ Evaluations	MloU (%)	MPA (%)	MPrecision (%)	MDice (%)	Hausdorf95 (mm)	ASD (mm)
UNetVggl6	68.3	78.96	81.04	79.87	52.87	1.99
HDC + UNetVggl6	77.81	84.57	89.75	86.9	25.79	0.49
Focal-Dice Loss + UNetVggl6	77.01	83.77	89.27	86.39	38.29	1.29

### UNetVGG16 + focal dice loss

After the validity of the HDC module was proven, we removed the HDC module and replaced the cross-entropy loss function in UNetVGG16 with the proposed focal Dice loss. The focal Dice loss weight was set to 4:1. As seen in Table 1, the use of the proposed focal Dice loss greatly improved, or reduced, the values of most performance indicators. MIoU and Hausdorf95 changed the most, with MIoU increasing by 9 percentage points and Hausdorf95 decreasing by 14.5 percentage points.

### SCU-Net

Finally, we use SCU-Net to carry out 50 iterations, and the initial learning rate is set to 10^−4^. The weight of the focal Dice loss function was set to 4:1. For UNetVGG16 and UNetResNet50, we use the weights pretrained by VGG16 and ResNet50 on the VOC dataset for migration learning. The initial learning rate is set to 10^−4^ in the freezing phase and 10^−5^ in the unfreezing phase. For U-Net, DeepLabv3ResNet50, and FCN8s, we directly conduct 50 iterations for training, and the initial learning rate is set as 10^−4^. Table 2 shows the performance of SCU-Net and several commonly used segmentation algorithms under different indices in the same tumour dataset. It can be seen in the figure that all indices of SCU-Net are ahead of other networks and have the best performance, followed by UNetVGG16 and the balanced performance of all indices, followed by FCN8s. U-Net and DeepLabv3ResNet50 perform poorly. They do not achieve good segmentation accuracy and classification accuracy.

**Table 2:** Performance comparison between Scu-Net and several commonly used partition networks

Networks \ Evalustions	MloU (%)	MPA (%)	MPrecision (%)	MDice (%)	Hausdorf95 (mm)	ASD (mm)
UNetVgg16	84.22	90.31	92.06	91.17	27.35	0.85
UNetResNet50	62.08	67.67	86.31	74.6	59.79	0.95
UNet	76.26	79.77	93.76	85.9	37.37	0.42
Deep1abv3ResNet50	69.48	79.5	82.58	80.82	44.01	1.07
FCN8s	81.16	84.72	94.52	89.23	30.09	0.74
SCU-Net (Ours)	86.8	90.74	94.63	92.62	17.71	0.37

**Fig. 3:**
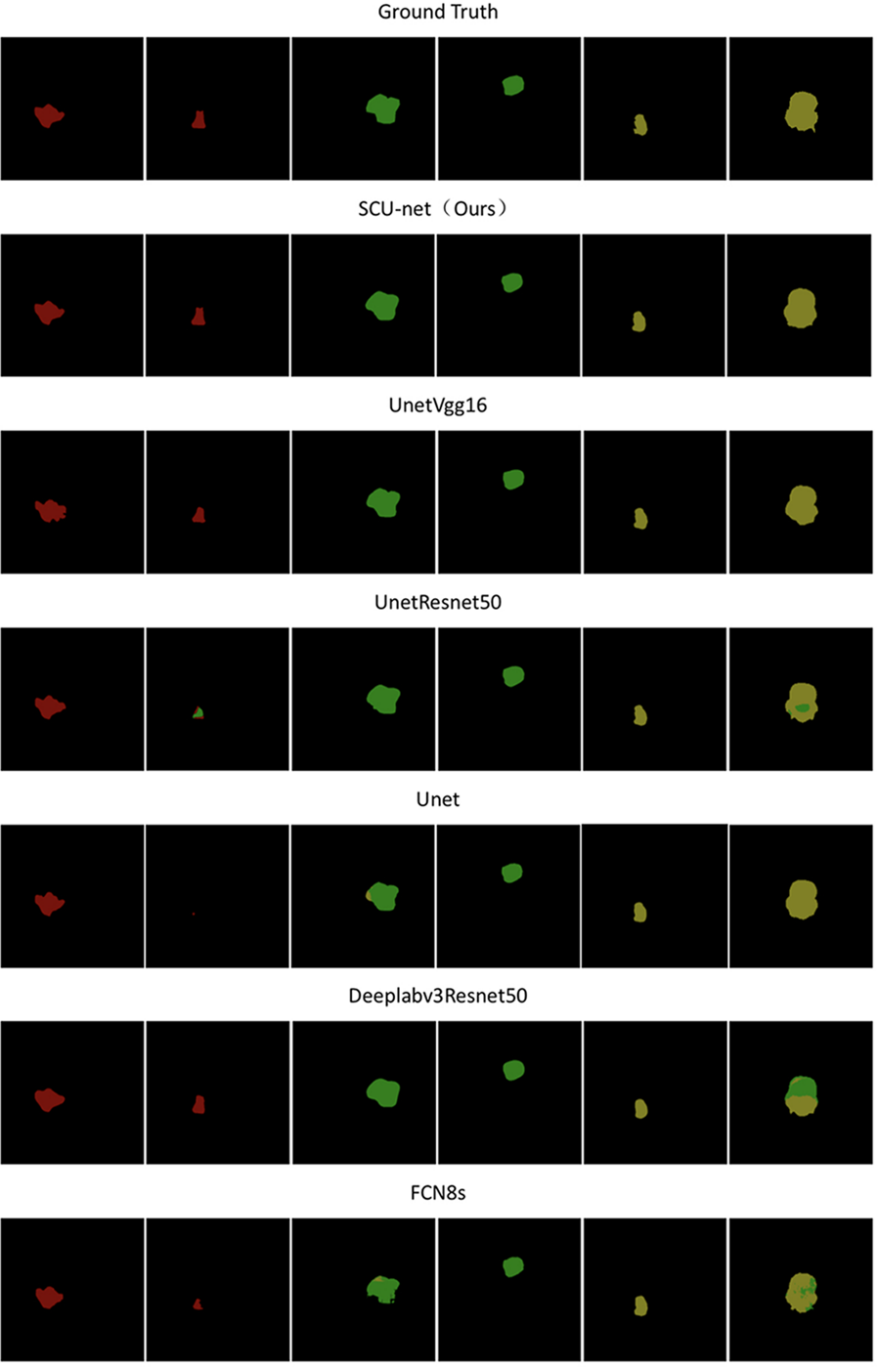
Comparison of segmentation results of different types of tumours by different networks

Figure 3 shows the comparison of the segmentation results and the ground truth of 6 different types of tumours by different models, in which the red region represents glioma tumours, the green region represents meningioma tumours, and the yellow region represents pituitary tumours. It can be seen in the figure that the first segmentation result of UNetVGG16 is quite different from the ground truth. The segmentation results of other classes by UNetVGG16 are basically consistent with the ground truth, but the edges are still too smooth, and the segmentation details of the edges are not ideal. Some glioma tumours are incorrectly predicted as meningioma tumours in the second segmentation figure of UNetResNet50, and the segmentation result is a triangle, which is quite different from the ground truth. The second segmentation result graph of U-Net almost does not segment the tumour, so the effect was poor. The last segmentation map of DeepLabv3ResNet50 has nearly 50% of the regions incorrectly classified, while the segmentation results of other segmentation maps are obviously too smooth, although the categories are correctly predicted. The effect of the second segmentation map of FCN8s is poor, which is quite different from the ground truth. The segmentation result of SCU-Net (Ours) is the best, there is almost no error prediction, and the edge segmentation effect is obviously better than other models. The experimental results show that SCU-Net has good robustness on the brain tumour dataset presented in this paper.

## Discussion

### UNetVGG16 + HDC

To verify whether UNetVGG16 with the HDC module can capture more detailed information, we compare UNetvGG16 with HDC with the original UNetVGG16. It can be seen in Table 1 that HDC + UNetVGG16 has great improvements in both segmentation ability and classification accuracy. This is because the HDC module enlarges the receptive field of the model and solves the problem of incomplete information capture caused by the traditional expanded convolution chessboard phenomenon so that the algorithm can better extract the characteristics of the image. Therefore, it is a good choice to use HDC in the backbone network of UNetVGG16, which can improve the model's classification accuracy and segmentation accuracy.

### UNetVGG16 + focal dice loss

To verify whether our proposed focal Dice loss can specifically learn samples that are difficult to segment and classify to improve the overall performance of the algorithm, we designed a comparative experiment between UNetVGG16 + focal Dice loss and UNetVGG16, as shown in Table 1. The experimental results show that the proposed focal Dice loss contributes to the segmentation network performance and greatly improves tumour recognition accuracy and segmentation performance. We accelerate the training of difficult training samples by increasing the loss weight of difficult segmentation and classification to improve the model performance, which means that we can better deal with brain tumour samples that are more difficult to train in the training set to effectively solve the problems of unbalanced tumour category samples and different qualities of tumour sample images.

### SCU-Net

Table 2 shows that our SCU-Net is different from U-Net and the other four commonly used segmentation algorithms. It can be seen in the table that SCU-Net has the best performance under most indicators, and its performance is far better than that of U-Net. Figure 3 shows the segmentation results of SCU-Net, U-Net and several other commonly used segmentation algorithms. It can be seen that the effect of SCU-Net is closest to the ground truth, and the detail processing is also better than the other algorithms. According to the experiment, it can be inferred that although FCN8s adopt 8 times upsampling, which is much better than 32 times, it still lacks details and is not sufficient to achieve a high segmentation effect. UNetResNet50 and DeepLabv3ResNet50 easily produce gradient disappearance and other problems because the backbone network model is too deep. The tumour images are composed of simple textures and shapes and the most typical features. If ResNet50 is used, it may cause feature redundancy, which affects the final result, so VGG16 as the SCU-Net backbone network is the best choice. Through serial operation, SCU-Net not only does not result in feature redundancy but also uses two decoding networks for feature extraction twice, as well as the fusion of features and pixels, which improves the semantic segmentation performance of the network.

## Conclusion

In this paper, we proposed an improved U-Net algorithm, called SCU-Net, for segmenting brain tumours. We operate two U-Net models with VGG16 as the backbone in tandem and perform feature splicing and decoding module splicing at each layer so that the two encoding–decoding blocks before and after can form a tighter connection to obtain more semantic information, reduce feature redundancy, and further improve the generalization ability of the model. Since location information is extremely important for brain tumour category classification, we add another HDC module to the U-Net encoding network to obtain a larger receptive field to capture more location information. In addition, the proposed focal Dice loss enables the model to consistently focus not only on pixel classification accuracy but also on segmentation performance during training and focus more on the samples that are difficult to classify and divide. We compared SCU-NET with commonly used brain tumour segmentation models under 6 indicators. Experimental results show that the proposed method can significantly improve target segmentation and tumour prediction performance.

## Data Availability

The data in this article adopt Kaggle's official open source dataset. Download address: https://www.kaggle.com/datasets/iashiqul/brain-tumor-mri-image-classification.
